# Typhoid Fever

**Published:** 1874-10

**Authors:** Will. T. T. Montgomery

**Affiliations:** Chicago


A MONTHLY RECORD OF

*Medicine, Surgery and the Collateral Sciences.*

EDITED BY J. ADAMS ALLEN, M.D., LL.D.; AND WALTER HAY, M.D.

VOL. XXXI. — OCTOBER, 1874. — NO. 10.

### Original Communications.

ARTICLE I.—*Typhoid Fever—Collation of* 112 *Cases.* By WILL. T. T. MONTGOMERY, M.D., Chicago.

I have endeavored, in the following paper, to present what has seemed to me to be points of most interest from a record of 112 cases of typhoid fever. These cases were treated during the months of September, October and November, 1872, in Cook County Hospital, in the service of Drs. Joseph P. Ross and Henry M. Lyman, attending physicians, and during my service as resident physician of that hospital. It is through the kindness of the attending physicians that I am enabled to present this report, and whatever of credit or merit attaches to the treatment of these cases belongs to these gentlemen.

I need not remind members of this Society who were practicing in the city in the fall of 1872, that there was an unusual prevalence of typhoid fever here at that time, but I think the number of cases admitted into the charity hospitals at that time was unusually large in proportion to the whole number of cases in the city, and the reasons are obvious.

*First.* While *epidemics* of typhoid are not common in Chicago, *cases* are not uncommon. Indeed, rarely a month passes that does not present a greater or less number.

*Second.* It is an established fact, that persons going from the country, or portions of it where typhoid fever is not endemic, into a place where it is endemic, are much more susceptible to the influence of the typhoid poison. In other words, the system becomes accommodated to the poison by being with it.

*Third.* A large number of the cases seeking admission into the charity hospitals are recent arrivals in the city.

*Fourth.* In the spring and summer of 1872 Chicago had an unprecedented ingathering of men from all parts of the country to engage in the great work of rebuilding the city.

I mention these as being to me the most prominent reasons why the proportion of cases in hospital was unusually large in the fall of 1872. It is not my purpose, in presenting this paper, to enter into a general discussion of the subject of typhoid fever, but to make a brief statement of the most marked symptoms, complications and sequelæ met with in these cases, notice the importance of each, and call attention to the treatment adopted. We will first give an epitome of these, and then take up each separately and dispose of it.

*Symptoms.* In the 112 cases, epistaxis occurred in 23; diarrhœa in 77; constipation in 10; rose spots in 50; blue spots in 2. In all of the cases particular attention was given to the temperature, pulse, and respirations.

*Complications.* Delirium occurred in 49; deafness in 11; softening of the cornea in 1; parotitis in 3; intestinal hemorrhage in 3; peritonitis in 2.

*Sequelæ.* Right hemiplegia, with aphasia, occurred in 1; œdema of left lower extremity in 3; died, 20.

We will next consider each of these separately.

*Epistaxis.* This, you will remember, occurred in 23 of 112 cases. In 20 of the 23, the epistaxis occurred in the first week, and in 3 in the third week of the disease. When it occurs in the first week, epistaxis is a symptom of much diagnostic importance; especially is this true when it occurs in a patient complaining of headache. Almost all of the 112 cases who were able to tell how their sickness began, stated that they first began to have headache

### Tabulated Statement of Twenty-Four Cases of Typhoid Fever.

| Cases | Day of Disease on which observation began | Pulse, Temperature, Respiration | 1st Day M. | 1st Day E. | 2d Day M. | 2d Day E. | 3d Day M. | 3d Day E. | 4th Day M. | 4th Day E. | 5th Day M. | 5th Day E. | 6th Day M. | 6th Day E. | 7th Day M. | 7th Day E. | 8th Day M. | 8th Day E. | 9th Day M. | 9th Day E. | 10th Day M. | 10th Day E. | 11th Day M. | 11th Day E. | 12th Day M. | 12th Day E. | 13th Day M. | 13th Day E. | 14th Day M. | 14th Day E. | 15th Day M. | 15th Day E. | 16th Day M. | 16th Day E. | 17th Day M. | 17th Day E. | 18th Day M. | 18th Day E. | 19th Day M. | 19th Day E. | 20th Day M. | 20th Day E. | 21st Day M. | 21st Day E. | 22d Day M. | 22d Day E. | 23d Day M. | 23d Day E. | 24th Day M. | 24th Day E. | 25th Day M. | 25th Day E. | 26th Day M. | 26th Day E. | 27th Day M. | 27th Day E. | 28th Day M. | 28th Day E. | Remarks. |
|---|---|---|---|---|---|---|---|---|---|---|---|---|---|---|---|---|---|---|---|---|---|---|---|---|---|---|---|---|---|---|---|---|---|---|---|---|---|---|---|---|---|---|---|---|---|---|---|---|---|---|---|---|---|---|---|---|---|---|---|
| 1 | 7th | P. | 86 | 96 | 88 | 84 | ---- | 112 | 96 | ---- | 92 | 100 | 92 | 90 | 96 | 124 | 104 | 116 | 100 | 108 | 108 | ---- | 108 | 106 | 104 | 90 | 96 | 128 | 90 | 92 | 100 | 108 | 90 | 116 | 92 | 96 | 100 | 98 | 94 | ---- | ---- | ---- | ---- | ---- | ---- | ---- | ---- | ---- | ---- | ---- | ---- | ---- | ---- | ---- | ---- | ---- | ---- | ---- | Œdema of left lower extremity. Recovered. |
| | | T. | 103 | 104.5 | 103 | 104 | ---- | 106.4 | 105.8 | ---- | 103 | 104.5 | 103 | 104.5 | 105 | 106.6 | 103 | 106 | 105.2 | 106.5 | 102.4 | ---- | 101 | 101.5 | 101 | 103.6 | 102.5 | 106 | 101 | 104 | 101.5 | 102.5 | 101.4 | 105.5 | 101 | 101.5 | 101 | 101 | 100 | ---- | ---- | ---- | ---- | ---- | ---- | ---- | ---- | ---- | ---- | ---- | ---- | ---- | ---- | ---- | ---- | ---- | ---- | ---- | |
| | | R. | 19 | 27 | 18 | 18 | ---- | 20 | 28 | ---- | 26 | 21 | 21 | 20 | 24 | 28 | 24 | 24 | 18 | 24 | 20 | ---- | 20 | 24 | 16 | 24 | 20 | 24 | 23 | 24 | 21 | 23 | 12 | 24 | 16 | 18 | 25 | 24 | 20 | ---- | ---- | ---- | ---- | ---- | ---- | ---- | ---- | ---- | ---- | ---- | ---- | ---- | ---- | ---- | ---- | ---- | ---- | ---- | |
| 2 | 6th | P. | 90 | 103 | 92 | 104 | 96 | 108 | 96 | 100 | 104 | 116 | 104 | 104 | 102 | ---- | 93 | 100 | 100 | 104 | 106 | 104 | 96 | ---- | 100 | 104 | 108 | 120 | 100 | 108 | 100 | 108 | 104 | 108 | 106 | 116 | 116 | 100 | 96 | 98 | 94 | 92 | ---- | ---- | ---- | ---- | ---- | ---- | ---- | ---- | ---- | ---- | ---- | ---- | ---- | ---- | ---- | ---- | Uncomplicated. Recovered. |
| | | T. | 104 | 106.5 | 104.5 | 105.5 | 104 | 105 | 104.5 | 103.6 | 102 | 105 | 102.6 | 106 | 104 | ---- | 102 | 104 | 101.5 | 105 | 102 | 104 | 102.5 | ---- | 103.5 | 105.4 | 102.5 | 105.3 | 101.5 | 105 | 101.5 | 105 | 101.3 | 104.4 | 103 | 105.5 | 102 | 105 | 101 | 104.3 | 100 | 102 | ---- | ---- | ---- | ---- | ---- | ---- | ---- | ---- | ---- | ---- | ---- | ---- | ---- | ---- | ---- | ---- | |
| | | R. | 30 | 38 | 32 | 37 | 25 | 28 | 30 | 27 | 32 | 28 | 25 | 32 | 28 | ---- | 27 | 28 | 24 | 28 | 32 | 36 | 25 | ---- | 20 | 28 | 28 | 32 | 24 | 30 | 28 | 30 | 28 | 30 | 32 | 32 | 36 | 30 | 24 | 25 | 22 | 24 | ---- | ---- | ---- | ---- | ---- | ---- | ---- | ---- | ---- | ---- | ---- | ---- | ---- | ---- | ---- | ---- | |
| 3 | 3d | P. | 80 | 92 | 96 | 92 | 96 | 92 | 104 | 98 | 108 | ---- | 84 | 104 | 104 | 108 | 104 | 104 | 96 | 84 | 100 | ---- | 84 | 84 | 96 | 84 | 75 | 98 | ---- | ---- | 72 | 84 | 69 | 70 | 84 | ---- | ---- | 80 | ---- | ---- | ---- | ---- | ---- | ---- | ---- | ---- | ---- | ---- | ---- | ---- | ---- | ---- | ---- | ---- | ---- | ---- | ---- | ---- | Parotitis and suppuration of both glands. Recovered. |
| | | T. | 100 | 103 | 101 | 105 | 103.5 | 102.5 | 102 | 103.5 | 102 | ---- | 97.5 | 105 | 103.5 | 105 | 102.6 | 104 | 103.5 | 104.5 | 101 | ---- | 101 | 103 | 101 | 104 | 99.5 | 106 | ---- | ---- | 99.5 | 102.5 | 101 | 102 | 100.5 | ---- | ---- | 100 | ---- | ---- | ---- | ---- | ---- | ---- | ---- | ---- | ---- | ---- | ---- | ---- | ---- | ---- | ---- | ---- | ---- | ---- | ---- | ---- | |
| | | R. | 20 | 20 | 26 | 16 | 32 | 20 | 20 | 24 | 24 | ---- | 20 | 18 | 28 | 24 | 23 | 24 | 20 | 30 | 27 | ---- | 27 | 25 | 20 | 20 | 21 | 28 | ---- | ---- | 18 | 32 | 20 | 23 | 20 | ---- | ---- | 20 | ---- | ---- | ---- | ---- | ---- | ---- | ---- | ---- | ---- | ---- | ---- | ---- | ---- | ---- | ---- | ---- | ---- | ---- | ---- | ---- | |
| 4 | 5th | P. | 94 | 104 | 96 | 96 | 80 | 96 | 96 | 84 | 70 | 88 | 80 | ---- | 76 | 92 | 80 | 80 | 96 | ---- | 92 | 92 | 88 | ---- | ---- | ---- | ---- | ---- | ---- | ---- | ---- | ---- | ---- | ---- | ---- | ---- | ---- | ---- | ---- | ---- | ---- | ---- | ---- | ---- | ---- | ---- | ---- | ---- | ---- | ---- | ---- | ---- | ---- | ---- | ---- | ---- | ---- | ---- | Uncomplicated. Recovered. |
| | | T. | 103 | 105 | 104 | 105 | 103 | 105 | 104 | 103 | 100.5 | 102.5 | 102 | ---- | 100 | 102 | 102 | 102.5 | 102 | ---- | 102 | 103 | 102 | ---- | ---- | ---- | ---- | ---- | ---- | ---- | ---- | ---- | ---- | ---- | ---- | ---- | ---- | ---- | ---- | ---- | ---- | ---- | ---- | ---- | ---- | ---- | ---- | ---- | ---- | ---- | ---- | ---- | ---- | ---- | ---- | ---- | ---- | ---- | |
| | | R. | 26 | 30 | 28 | 24 | 24 | 25 | 20 | 24 | 20 | 23 | 18 | ---- | 16 | 20 | 18 | 20 | 20 | ---- | 20 | 20 | 20 | ---- | ---- | ---- | ---- | ---- | ---- | ---- | ---- | ---- | ---- | ---- | ---- | ---- | ---- | ---- | ---- | ---- | ---- | ---- | ---- | ---- | ---- | ---- | ---- | ---- | ---- | ---- | ---- | ---- | ---- | ---- | ---- | ---- | ---- | ---- | |
| 5 | 16th | P. | 108 | 120 | 100 | 108 | 120 | 128 | 102 | 120 | 104 | 120 | 94 | 108 | 90 | 104 | 92 | ---- | 84 | ---- | 84 | 108 | 88 | 104 | ---- | ---- | ---- | ---- | ---- | ---- | ---- | ---- | ---- | ---- | ---- | ---- | ---- | ---- | ---- | ---- | ---- | ---- | ---- | ---- | ---- | ---- | ---- | ---- | ---- | ---- | ---- | ---- | ---- | ---- | ---- | ---- | ---- | ---- | Severe Bronchitis. Recovered. |
| | | T. | 105.5 | 105 | 105 | 105 | 104.5 | 106 | 104.5 | 105.5 | 102.5 | 105.4 | 101 | 103.5 | 101 | 104.5 | 101 | ---- | 101 | ---- | 100 | 103 | 99 | 103 | ---- | ---- | ---- | ---- | ---- | ---- | ---- | ---- | ---- | ---- | ---- | ---- | ---- | ---- | ---- | ---- | ---- | ---- | ---- | ---- | ---- | ---- | ---- | ---- | ---- | ---- | ---- | ---- | ---- | ---- | ---- | ---- | ---- | ---- | |
| | | R. | 32 | 36 | 36 | 40 | 40 | 36 | 40 | 32 | 32 | 38 | 32 | 44 | 40 | 40 | 30 | ---- | 36 | ---- | 24 | 28 | 24 | 30 | ---- | ---- | ---- | ---- | ---- | ---- | ---- | ---- | ---- | ---- | ---- | ---- | ---- | ---- | ---- | ---- | ---- | ---- | ---- | ---- | ---- | ---- | ---- | ---- | ---- | ---- | ---- | ---- | ---- | ---- | ---- | ---- | ---- | ---- | |
| 6 | 11th | P. | 100 | ---- | 96 | 112 | 104 | 116 | 112 | 108 | 116 | 124 | 100 | 112 | ---- | ---- | 100 | 108 | 96 | 120 | 100 | 96 | 100 | 104 | 112 | 100 | 116 | 90 | 100 | 80 | 108 | 84 | 99 | 96 | 90 | 92 | ---- | ---- | ---- | ---- | ---- | ---- | ---- | ---- | ---- | ---- | ---- | ---- | ---- | ---- | ---- | ---- | ---- | ---- | ---- | ---- | ---- | ---- | Severe Bronchitis. Recovered. |
| | | T. | 105.5 | ---- | 103.4 | 105 | 103 | 105 | 104.5 | 104.6 | 104.5 | 106 | 102 | 106 | ---- | ---- | 102 | 103.5 | 99 | 104.5 | 103 | 103 | 102 | 102 | 101.5 | 103 | 104 | 102.6 | 104.3 | 102 | 105 | 100 | 102 | 100 | 100 | 101 | ---- | ---- | ---- | ---- | ---- | ---- | ---- | ---- | ---- | ---- | ---- | ---- | ---- | ---- | ---- | ---- | ---- | ---- | ---- | ---- | ---- | ---- | |
| | | R. | 30 | ---- | 28 | 26 | 32 | 32 | 32 | 36 | 28 | 36 | 26 | 40 | ---- | ---- | 22 | 38 | 28 | 28 | 30 | 28 | 30 | 22 | 32 | 28 | 36 | 28 | 36 | 28 | 36 | 24 | 20 | 23 | 22 | 20 | ---- | ---- | ---- | ---- | ---- | ---- | ---- | ---- | ---- | ---- | ---- | ---- | ---- | ---- | ---- | ---- | ---- | ---- | ---- | ---- | ---- | ---- | |
| 7 | 4th | P. | 104 | ---- | 108 | 120 | 104 | 124 | 112 | 120 | 112 | 104 | 108 | 116 | ---- | ---- | 104 | 100 | 96 | 116 | 108 | 112 | 108 | 108 | 104 | 96 | 92 | 104 | 100 | 120 | ---- | ---- | 96 | 104 | 90 | 94 | 86 | 90 | ---- | ---- | ---- | ---- | ---- | ---- | ---- | ---- | ---- | ---- | ---- | ---- | ---- | ---- | ---- | ---- | ---- | ---- | ---- | ---- | Uncomplicated. Recovered. |
| | | T. | 103 | ---- | 103.7 | 104 | 102 | 105 | 103 | 104 | 103.5 | 105 | 103.4 | 105 | ---- | ---- | 103 | 104.5 | 102.6 | 104 | 104 | 105 | 103 | 105 | 103 | 105 | 102.5 | 104.3 | 102 | 104.5 | ---- | ---- | 103 | 105 | 102 | 103 | 101 | 101 | ---- | ---- | ---- | ---- | ---- | ---- | ---- | ---- | ---- | ---- | ---- | ---- | ---- | ---- | ---- | ---- | ---- | ---- | ---- | ---- | |
| | | R. | 24 | ---- | 21 | 34 | 28 | 26 | 24 | 28 | 24 | 28 | 32 | 28 | ---- | ---- | 24 | 23 | 20 | 24 | 24 | 26 | 28 | 36 | 30 | 24 | 24 | 22 | 20 | 32 | ---- | ---- | 30 | 32 | 24 | 26 | 20 | 22 | ---- | ---- | ---- | ---- | ---- | ---- | ---- | ---- | ---- | ---- | ---- | ---- | ---- | ---- | ---- | ---- | ---- | ---- | ---- | ---- | |
| 8 | 9th | P. | 104 | 100 | 93 | 116 | 112 | 104 | 100 | 104 | 92 | 108 | 92 | 96 | 100 | ---- | 98 | 100 | 96 | 96 | 92 | 108 | 96 | 104 | 112 | 104 | 112 | ---- | 104 | 120 | 108 | 116 | 104 | 116 | 108 | 104 | 104 | 108 | 96 | 124 | 112 | 104 | 96 | 108 | 100 | 108 | 112 | 128 | 124 | 120 | 128 | 120 | 116 | 160 | 156 | ---- | ---- | ---- | Hemorrhage from bowels. Death. |
| | | T. | 104.7 | 105 | 103.8 | 107 | 105 | 107 | 104.5 | 104 | 102.5 | 107.2 | 105.4 | 106 | 104 | ---- | 101.5 | 104.5 | 102 | 106 | 101 | 104.5 | 102 | 105.5 | 104.4 | 105.5 | 102.6 | ---- | 104 | 106 | 104.5 | 105.5 | 104.6 | 106 | 104 | 106 | 103.5 | 106 | 102.5 | 106.5 | 103 | 104.5 | 102 | 105 | 103.5 | 102.5 | 105 | 106.3 | 104 | 104.3 | 101.5 | 105 | 103 | 106.5 | 101.5 | ---- | ---- | ---- | |
| | | R. | 24 | 26 | 24 | 28 | 24 | 22 | 24 | 28 | 36 | 28 | 32 | 30 | 24 | ---- | 32 | 36 | 32 | 32 | 32 | 40 | 26 | 32 | 40 | 30 | 36 | ---- | 36 | 40 | 32 | 40 | 36 | 36 | 36 | 40 | 28 | 32 | 18 | 36 | 28 | 24 | 16 | 20 | 20 | 38 | 28 | 40 | 30 | 28 | 36 | 28 | 32 | 40 | 52 | ---- | ---- | ---- | |
| 9 | 14th | P. | ---- | 84 | 80 | 72 | 92 | 96 | 92 | 94 | 72 | 84 | 96 | ---- | 80 | 96 | 100 | 92 | 88 | ---- | 90 | 84 | 76 | 76 | 78 | 74 | ---- | ---- | ---- | ---- | ---- | ---- | ---- | ---- | ---- | ---- | ---- | ---- | ---- | ---- | ---- | ---- | ---- | ---- | ---- | ---- | ---- | ---- | ---- | ---- | ---- | ---- | ---- | ---- | ---- | ---- | ---- | ---- | Uncomplicated. Recovered. |
| | | T. | ---- | 106.2 | 104.5 | 105 | 104 | 105 | 106 | 106 | 103.5 | 104 | 104.5 | ---- | 102.6 | 105.5 | 103 | 104.5 | 102 | ---- | 101.5 | 105.5 | 101.5 | 103 | 101 | 102 | ---- | ---- | ---- | ---- | ---- | ---- | ---- | ---- | ---- | ---- | ---- | ---- | ---- | ---- | ---- | ---- | ---- | ---- | ---- | ---- | ---- | ---- | ---- | ---- | ---- | ---- | ---- | ---- | ---- | ---- | ---- | ---- | |
| | | R. | ---- | 28 | 32 | 24 | 20 | 24 | 24 | 26 | 24 | 20 | 18 | ---- | 18 | 20 | 24 | 20 | 24 | ---- | 28 | 28 | 18 | 28 | 20 | 22 | ---- | ---- | ---- | ---- | ---- | ---- | ---- | ---- | ---- | ---- | ---- | ---- | ---- | ---- | ---- | ---- | ---- | ---- | ---- | ---- | ---- | ---- | ---- | ---- | ---- | ---- | ---- | ---- | ---- | ---- | ---- | ---- | |
| 10 | 15th | P. | 100 | 112 | ---- | ---- | 108 | 120 | 116 | 108 | 116 | ---- | 90 | 120 | 92 | 108 | 92 | 120 | 116 | 110 | 104 | 104 | 108 | 96 | 100 | ---- | 104 | 120 | 96 | 114 | 104 | 108 | 104 | 112 | 108 | ---- | 112 | ---- | ---- | ---- | ---- | ---- | ---- | ---- | ---- | ---- | ---- | ---- | ---- | ---- | ---- | ---- | ---- | ---- | ---- | ---- | ---- | ---- | Uncomplicated. Recovered. |
| | | T. | 101 | 105 | ---- | ---- | 104.5 | 105 | 104 | 104.5 | 103.5 | ---- | 104 | 104 | 102 | 104 | 102.5 | 104 | 103 | 103.3 | 102.5 | 103.5 | 101 | 103 | 100 | ---- | 101 | 103 | 102 | 104 | 101 | 102 | 100 | 103 | 102 | ---- | 101 | ---- | ---- | ---- | ---- | ---- | ---- | ---- | ---- | ---- | ---- | ---- | ---- | ---- | ---- | ---- | ---- | ---- | ---- | ---- | ---- | ---- | |
| | | R. | 18 | 20 | ---- | ---- | 24 | 23 | 20 | 28 | 24 | ---- | 24 | 28 | 28 | 32 | 25 | 30 | 24 | 32 | 28 | 28 | 24 | 24 | 24 | ---- | 24 | 20 | 24 | 36 | 24 | 24 | 24 | 30 | 30 | ---- | 26 | ---- | ---- | ---- | ---- | ---- | ---- | ---- | ---- | ---- | ---- | ---- | ---- | ---- | ---- | ---- | ---- | ---- | ---- | ---- | ---- | ---- | |
| 11 | 3d | P. | 80 | 82 | 80 | 96 | 84 | 92 | 90 | 96 | 92 | 108 | 90 | 94 | 80 | 92 | 96 | 88 | 84 | 84 | 92 | 94 | 90 | 92 | 88 | 90 | 86 | 90 | 84 | 86 | ---- | ---- | ---- | ---- | ---- | ---- | ---- | ---- | ---- | ---- | ---- | ---- | ---- | ---- | ---- | ---- | ---- | ---- | ---- | ---- | ---- | ---- | ---- | ---- | ---- | ---- | ---- | ---- | Uncomplicated. Recovered. |
| | | T. | 102 | 103 | 103 | 104.5 | 102 | 105 | 102 | 105 | 103 | 105 | 102 | 105 | 103 | 104.5 | 103 | 105 | 103 | 104.5 | 102 | 104 | 101 | 104 | 100 | 106 | 99.5 | 104 | 100 | 101 | ---- | ---- | ---- | ---- | ---- | ---- | ---- | ---- | ---- | ---- | ---- | ---- | ---- | ---- | ---- | ---- | ---- | ---- | ---- | ---- | ---- | ---- | ---- | ---- | ---- | ---- | ---- | ---- | |
| | | R. | 18 | 20 | 17 | 20 | 20 | 24 | 16 | 16 | 20 | 28 | 20 | 20 | 20 | 20 | 22 | 16 | 18 | 20 | 20 | 18 | 20 | 20 | 18 | 24 | 18 | 18 | 20 | 18 | ---- | ---- | ---- | ---- | ---- | ---- | ---- | ---- | ---- | ---- | ---- | ---- | ---- | ---- | ---- | ---- | ---- | ---- | ---- | ---- | ---- | ---- | ---- | ---- | ---- | ---- | ---- | ---- | |
| 12 | 12th | P. | ---- | 92 | 90 | 104 | 90 | 96 | 92 | 104 | ---- | ---- | ---- | ---- | 100 | ---- | 104 | 116 | 100 | 124 | 92 | 116 | 96 | 104 | 104 | ---- | 108 | ---- | 106 | ---- | 96 | 100 | 104 | 104 | 120 | 104 | 104 | 104 | 102 | 92 | 100 | ---- | 90 | ---- | 96 | 96 | 90 | 100 | ---- | ---- | ---- | ---- | ---- | ---- | ---- | ---- | ---- | ---- | Deafness. Recovered. |
| | | T. | ---- | 104.5 | 103.4 | 105 | 104 | 104.5 | 102 | 104 | ---- | ---- | ---- | ---- | 102 | ---- | 105 | 106 | 103.5 | 106.5 | 103 | 104.5 | 102 | 106 | 104 | ---- | 102 | ---- | 103 | ---- | 104 | 104 | 104.3 | 101.5 | 101 | 101 | 102 | 102.5 | 99 | 98.5 | 99 | ---- | 102 | ---- | 100 | 101 | 99.5 | 100.5 | ---- | ---- | ---- | ---- | ---- | ---- | ---- | ---- | ---- | ---- | |
| | | R. | ---- | 28 | 28 | 40 | 32 | 28 | 25 | 24 | ---- | ---- | ---- | ---- | 28 | ---- | 30 | 36 | 28 | 40 | 24 | 44 | 40 | 30 | 36 | ---- | 24 | ---- | 24 | ---- | 20 | 40 | 24 | 32 | 56 | 32 | 28 | 28 | 24 | 28 | 28 | ---- | 18 | ---- | 18 | 18 | 18 | 20 | ---- | ---- | ---- | ---- | ---- | ---- | ---- | ---- | ---- | ---- | |
| 13 | 4th | P. | ---- | 90 | 80 | ---- | 68 | 80 | 80 | 96 | 72 | 96 | 84 | 80 | 72 | 80 | 80 | 80 | 80 | 80 | 90 | 80 | 80 | 84 | 80 | 96 | 92 | 78 | 76 | 72 | 76 | 84 | 76 | 84 | 76 | 80 | 80 | 84 | 72 | 92 | 72 | 84 | ---- | 92 | 92 | 80 | 70 | ---- | 76 | 88 | 80 | 76 | 80 | 84 | 72 | 84 | 84 | 72 | Right Hemiplegia and Aphasia. Recovered. |
| | | T. | ---- | 104.5 | 102.5 | ---- | 103 | 104 | 102 | 106 | 102.5 | 105 | 104 | 105 | 104 | 105 | 103.7 | 105 | 104 | 105 | 104 | 105 | 103 | 105 | 103 | 104.5 | 102 | 102.5 | 100.5 | 102 | 100.7 | 101.5 | 101 | 104.5 | 100 | 103 | 101.3 | 102.5 | 99.5 | 103.6 | 100 | 103.5 | ---- | 103.2 | 103.5 | 104.5 | 102 | ---- | 101.5 | 102.5 | 102 | 104 | 99.5 | 102 | 100 | 101 | 100.5 | 102 | |
| | | R. | ---- | 20 | 18 | ---- | 18 | 20 | 16 | 24 | 20 | 26 | 18 | 16 | 19 | 20 | 14 | 20 | 21 | 18 | 16 | 16 | 14 | 20 | 16 | 20 | 20 | 20 | 18 | 20 | 16 | 20 | 20 | 14 | 16 | 14 | 16 | 18 | 16 | 20 | 20 | 20 | ---- | 20 | 20 | 20 | 18 | ---- | 20 | 20 | 28 | 20 | 20 | 22 | 16 | 16 | 20 | 18 | |
| 14 | 14th | P. | ---- | 112 | 84 | 100 | 90 | 96 | 100 | 98 | 92 | 92 | 92 | 100 | 84 | 92 | 94 | ---- | 90 | 80 | 104 | 96 | 120 | 116 | 96 | 104 | 104 | 90 | 96 | ---- | 96 | 96 | 104 | 106 | 104 | 96 | 106 | 96 | 100 | ---- | 78 | 136 | 120 | 130 | 140 | 150 | ---- | ---- | ---- | ---- | ---- | ---- | ---- | ---- | ---- | ---- | ---- | ---- | Peritonitis. Death. |
| | | T. | ---- | 104.5 | 103 | 104 | 102.5 | 104.3 | 103 | 104 | 103 | 104.5 | 103 | 105.6 | 103.5 | 105 | 103 | ---- | 101.5 | 104 | 105.4 | 102.5 | 103 | 105 | 102 | 103.5 | 101 | 103 | 101 | ---- | 102.3 | 103.5 | 103.8 | 103.5 | 101.5 | 103 | 103 | 102.5 | 101.3 | ---- | 104 | 104 | 104 | 104 | 103.5 | 104.5 | ---- | ---- | ---- | ---- | ---- | ---- | ---- | ---- | ---- | ---- | ---- | ---- | |
| | | R. | ---- | 23 | 16 | 24 | 20 | 18 | 16 | 20 | 18 | 14 | 16 | 18 | 18 | 16 | 14 | ---- | 20 | 20 | 28 | 20 | 20 | 28 | 20 | 24 | 23 | 20 | 24 | ---- | 24 | 24 | 26 | 24 | 24 | 18 | 18 | 24 | 24 | ---- | 20 | 40 | 36 | 28 | 36 | 52 | ---- | ---- | ---- | ---- | ---- | ---- | ---- | ---- | ---- | ---- | ---- | ---- | |
| 15 | 3d | P. | 104 | 108 | 104 | 120 | 112 | 132 | 108 | 120 | 116 | ---- | 112 | 118 | 116 | 132 | 96 | 120 | 120 | 116 | 116 | 116 | 128 | 130 | ---- | 136 | 120 | 124 | 132 | 120 | 132 | 104 | 120 | 124 | 108 | 120 | 104 | ---- | ---- | ---- | ---- | ---- | ---- | ---- | ---- | ---- | ---- | ---- | ---- | ---- | ---- | ---- | ---- | ---- | ---- | ---- | ---- | ---- | Phthisis complicating. |
| | | T. | 101 | 102.5 | 102 | 105 | 104 | 104.8 | 103 | 105 | 104 | ---- | 102.5 | 105 | 104.5 | 105 | 102.6 | 105 | 103 | 105 | 103 | 105.3 | 104 | 105 | ---- | 106 | 103 | 106.5 | 103.5 | 104.5 | 102 | 105 | 102 | 104 | 101.5 | 104 | 102 | ---- | ---- | ---- | ---- | ---- | ---- | ---- | ---- | ---- | ---- | ---- | ---- | ---- | ---- | ---- | ---- | ---- | ---- | ---- | ---- | ---- | |
| | | R. | 24 | 26 | 30 | 20 | 28 | 30 | 28 | 30 | 26 | ---- | 24 | 24 | 28 | 20 | 24 | 28 | 32 | 32 | 30 | 26 | 32 | 36 | ---- | 40 | 32 | 32 | 34 | 32 | 32 | 28 | 28 | 30 | 26 | 28 | 28 | ---- | ---- | ---- | ---- | ---- | ---- | ---- | ---- | ---- | ---- | ---- | ---- | ---- | ---- | ---- | ---- | ---- | ---- | ---- | ---- | ---- | |
| 16 | 10th | P. | ---- | 100 | 100 | 104 | 100 | 120 | 98 | ---- | 116 | 116 | 108 | 104 | 96 | 118 | 116 | ---- | 92 | 120 | 100 | 108 | 100 | 96 | 96 | 96 | 96 | 100 | 96 | 90 | 96 | ---- | 92 | 110 | 90 | 112 | 100 | 100 | 88 | 80 | 80 | ---- | 78 | 80 | 88 | ---- | 90 | 96 | 72 | 76 | 74 | 74 | ---- | ---- | ---- | ---- | ---- | ---- | Deafness. Recovered. |
| | | T. | ---- | 105 | 103.5 | 104.6 | 104 | 105 | 103.5 | ---- | 103.5 | 105.6 | 104.8 | 105.5 | 103.6 | 106 | 104.5 | ---- | 103.5 | 105 | 104 | 105 | 104 | 104 | 103.7 | 104 | 103 | 103.5 | 103.5 | 104.5 | 102 | ---- | 101 | 103 | 102.5 | 103 | 101.5 | 102.5 | 101.5 | 102.8 | 102.5 | ---- | 101 | 101.5 | 99.5 | ---- | 99 | 101 | 100 | 100.5 | 98.5 | 99 | ---- | ---- | ---- | ---- | ---- | ---- | |
| | | R. | ---- | 30 | 20 | 24 | 20 | 32 | 25 | ---- | 20 | 26 | 34 | 32 | 32 | 20 | 24 | ---- | 28 | 28 | 30 | 28 | 28 | 28 | 30 | 24 | 32 | 24 | 28 | 20 | 28 | ---- | 24 | 24 | 26 | 24 | 24 | 28 | 24 | 28 | 24 | ---- | 20 | 18 | 20 | ---- | 20 | 20 | 22 | 20 | 18 | 18 | ---- | ---- | ---- | ---- | ---- | ---- | |
| 17 | 8th | P. | ---- | 96 | 92 | 100 | 96 | ---- | 88 | 100 | 92 | 100 | 90 | 100 | 96 | 92 | 96 | 100 | 104 | 112 | 112 | ---- | 116 | 130 | 92 | 102 | 124 | 128 | 132 | 117 | 96 | 104 | 106 | 124 | 120 | ---- | 116 | 132 | 120 | 124 | 128 | 120 | 114 | 92 | 160 | ---- | ---- | ---- | ---- | ---- | ---- | ---- | ---- | ---- | ---- | ---- | ---- | ---- | Parotitis of right gland. Recovered. |
| | | T. | ---- | 104 | 102.8 | 103.5 | 102 | ---- | 102.5 | 103.5 | 103 | 104.5 | 100 | 104.5 | 102.7 | 105 | 103 | 105 | 103 | 102.5 | 100.5 | ---- | 100 | 101.5 | 99.5 | 99 | 101 | 102.5 | 102 | 101.5 | 99.5 | 100 | 100 | 102.5 | 101.5 | ---- | 103 | 104 | 102 | 102 | 101.5 | 103.5 | 103.5 | 102 | 103.5 | ---- | ---- | ---- | ---- | ---- | ---- | ---- | ---- | ---- | ---- | ---- | ---- | ---- | |
| | | R. | ---- | 32 | 28 | 26 | 28 | ---- | 24 | 24 | 24 | 28 | 28 | 24 | 28 | 30 | 24 | 28 | 24 | 24 | 28 | ---- | 30 | 28 | 28 | 26 | 28 | 28 | 28 | 26 | 18 | 24 | 20 | 28 | 20 | ---- | 32 | 30 | 24 | 28 | 32 | 30 | 32 | 40 | 76 | ---- | ---- | ---- | ---- | ---- | ---- | ---- | ---- | ---- | ---- | ---- | ---- | ---- | |
| 18 | 2d | P. | 80 | 80 | 80 | 80 | 84 | 80 | 80 | 82 | 78 | 80 | 84 | 96 | 92 | 92 | 72 | 96 | 100 | 88 | 88 | 80 | 88 | 80 | 80 | 100 | 84 | 92 | 80 | 88 | 80 | 72 | 80 | 96 | 81 | 92 | 76 | 74 | ---- | ---- | 88 | 86 | 96 | 80 | 88 | 90 | 94 | 84 | ---- | ---- | ---- | ---- | ---- | ---- | ---- | ---- | ---- | ---- | Mild. Recovered. |
| | | T. | 99.5 | 101 | 101 | 103 | 102 | 103 | 101 | 102.5 | 100 | 102 | 102 | 103.5 | 101 | 103.5 | 100 | 103 | 102 | 104 | 101.5 | 102 | 100.5 | 103 | 100.5 | 104.8 | 102 | 104.5 | 99.5 | 104.2 | 99.8 | 103.5 | 99 | 103.7 | 98.5 | 103 | 98.7 | 102 | ---- | ---- | 99.5 | 102.5 | 98.5 | 102 | 99 | 100 | 98.5 | 99 | ---- | ---- | ---- | ---- | ---- | ---- | ---- | ---- | ---- | ---- | |
| | | R. | 20 | 20 | 22 | 20 | 20 | 24 | 30 | 26 | 24 | 24 | 24 | 32 | 28 | 28 | 24 | 28 | 24 | 28 | 30 | 28 | 24 | 28 | 30 | 32 | 24 | 28 | 28 | 24 | 24 | 24 | 20 | 22 | 20 | 23 | 20 | 18 | ---- | ---- | 20 | 21 | 24 | 20 | 23 | 24 | 20 | 18 | ---- | ---- | ---- | ---- | ---- | ---- | ---- | ---- | ---- | ---- | |
| 19 | 5th | P. | 116 | 108 | 100 | 100 | 100 | 116 | 112 | 104 | 92 | 92 | 124 | ---- | ---- | ---- | 120 | 132 | 120 | 108 | 120 | 134 | 124 | ---- | 108 | 132 | 106 | 128 | 112 | 120 | 124 | 140 | 120 | 120 | 100 | 112 | 108 | ---- | 104 | 108 | 104 | 100 | 104 | 96 | 84 | 80 | ---- | ---- | ---- | ---- | ---- | ---- | ---- | ---- | ---- | ---- | ---- | ---- | Bronchitis. Recovered. |
| | | T. | 103.5 | 104 | 102 | 103.6 | 103 | 104 | 102.5 | 103 | 102.5 | 103 | 104.8 | ---- | ---- | ---- | 102.5 | 104.8 | 103 | 105 | 103.5 | 105 | 102 | ---- | 103.3 | 104.3 | 101 | 103 | 100 | 104 | 102.5 | 104.2 | 101 | 102 | 99 | 101 | 100 | ---- | 98.7 | 101 | 100 | 100.5 | 100 | 99.5 | 99 | 98.5 | ---- | ---- | ---- | ---- | ---- | ---- | ---- | ---- | ---- | ---- | ---- | ---- | |
| | | R. | 32 | 36 | 40 | 30 | 26 | 24 | 32 | 32 | 36 | 38 | 36 | ---- | ---- | ---- | 28 | 36 | 34 | 28 | 32 | 28 | 38 | ---- | 32 | 30 | 32 | 28 | 32 | 32 | 36 | 32 | 28 | 36 | 28 | 28 | 32 | ---- | 30 | 30 | 24 | 30 | 28 | 24 | 20 | 18 | ---- | ---- | ---- | ---- | ---- | ---- | ---- | ---- | ---- | ---- | ---- | ---- | |
| 20 | 6th | P. | ---- | 96 | 84 | 72 | 72 | ---- | 76 | 70 | 80 | 88 | 72 | 86 | 60 | 68 | 64 | ---- | 86 | 80 | 72 | 84 | 82 | 108 | 84 | 80 | 92 | 80 | 86 | 72 | 92 | 90 | 80 | 72 | ---- | ---- | ---- | ---- | ---- | ---- | ---- | ---- | ---- | ---- | ---- | ---- | ---- | ---- | ---- | ---- | ---- | ---- | ---- | ---- | ---- | ---- | ---- | ---- | Mild. Recovered. |
| | | T. | ---- | 105 | 103 | 103 | 102 | ---- | 104 | 104 | 103 | 104.5 | 103.5 | 104.5 | 101.5 | 103 | 102 | ---- | 101.5 | 105 | 100.5 | 103.8 | 100.5 | 104.5 | 100.5 | 104 | 101.5 | 102 | 100 | 101 | 100 | 100 | 100 | 100.5 | ---- | ---- | ---- | ---- | ---- | ---- | ---- | ---- | ---- | ---- | ---- | ---- | ---- | ---- | ---- | ---- | ---- | ---- | ---- | ---- | ---- | ---- | ---- | ---- | |
| | | R. | ---- | 22 | 23 | 20 | 20 | ---- | 22 | 20 | 24 | 24 | 20 | 20 | 24 | 23 | 25 | ---- | 23 | 24 | 18 | 20 | 19 | 28 | 22 | 20 | 23 | 20 | 24 | 18 | 24 | 21 | 20 | 18 | ---- | ---- | ---- | ---- | ---- | ---- | ---- | ---- | ---- | ---- | ---- | ---- | ---- | ---- | ---- | ---- | ---- | ---- | ---- | ---- | ---- | ---- | ---- | ---- | |
| 21 | 8th | P. | 100 | 98 | 100 | 116 | 116 | 120 | 120 | 120 | 120 | 128 | 120 | 132 | 142 | 160 | ---- | ---- | ---- | ---- | ---- | ---- | ---- | ---- | ---- | ---- | ---- | ---- | ---- | ---- | ---- | ---- | ---- | ---- | ---- | ---- | ---- | ---- | ---- | ---- | ---- | ---- | ---- | ---- | ---- | ---- | ---- | ---- | ---- | ---- | ---- | ---- | ---- | ---- | ---- | ---- | ---- | ---- | Uncomplicated. Bath used. Died. |
| | | T. | 105.7 | 106 | 105.5 | 107 | 106.5 | 106.5 | 104.5 | 106 | 104 | 106.5 | 103 | 106 | 105 | 105 | ---- | ---- | ---- | ---- | ---- | ---- | ---- | ---- | ---- | ---- | ---- | ---- | ---- | ---- | ---- | ---- | ---- | ---- | ---- | ---- | ---- | ---- | ---- | ---- | ---- | ---- | ---- | ---- | ---- | ---- | ---- | ---- | ---- | ---- | ---- | ---- | ---- | ---- | ---- | ---- | ---- | ---- | |
| | | R. | 28 | 26 | 24 | 28 | 28 | 24 | 28 | 24 | 30 | 28 | 28 | 30 | 48 | 48 | ---- | ---- | ---- | ---- | ---- | ---- | ---- | ---- | ---- | ---- | ---- | ---- | ---- | ---- | ---- | ---- | ---- | ---- | ---- | ---- | ---- | ---- | ---- | ---- | ---- | ---- | ---- | ---- | ---- | ---- | ---- | ---- | ---- | ---- | ---- | ---- | ---- | ---- | ---- | ---- | ---- | ---- | |
| 22 | 13th | P. | ---- | 100 | 108 | 96 | 80 | ---- | 72 | 100 | 92 | 96 | 84 | 108 | 84 | 108 | 92 | 112 | 104 | ---- | 104 | 116 | 96 | 116 | 96 | 108 | 88 | 104 | 92 | 104 | 92 | 104 | 96 | 100 | ---- | ---- | 96 | 100 | ---- | ---- | ---- | ---- | ---- | ---- | ---- | ---- | ---- | ---- | ---- | ---- | ---- | ---- | ---- | ---- | ---- | ---- | ---- | ---- | Uncomplicated. Recovered. |
| | | T. | ---- | 104 | 102 | 103 | 102.5 | ---- | 102 | 105 | 102 | 104.5 | 101 | 105.6 | 101 | 106 | 104 | 106 | 102 | ---- | 102.5 | 105.3 | 102 | 105.5 | 101 | 104.5 | 101 | 104 | 100.5 | 104.5 | 100.5 | 105.6 | 101 | 104.5 | ---- | ---- | 100 | 100.5 | ---- | ---- | ---- | ---- | ---- | ---- | ---- | ---- | ---- | ---- | ---- | ---- | ---- | ---- | ---- | ---- | ---- | ---- | ---- | ---- | |
| | | R. | ---- | 30 | 30 | 28 | 24 | ---- | 24 | 28 | 24 | 30 | 24 | 32 | 24 | 28 | 20 | 28 | 28 | ---- | 24 | 32 | 20 | 20 | 28 | 28 | 24 | 25 | 21 | 24 | 20 | 28 | 20 | 26 | ---- | ---- | 21 | 24 | ---- | ---- | ---- | ---- | ---- | ---- | ---- | ---- | ---- | ---- | ---- | ---- | ---- | ---- | ---- | ---- | ---- | ---- | ---- | ---- | |
| 23 | 5th | P. | ---- | 103 | 92 | 104 | 96 | 108 | 100 | 96 | 104 | 116 | 104 | 104 | 102 | ---- | 93 | 110 | 104 | 108 | 100 | 104 | ---- | ---- | 106 | 104 | 96 | ---- | 100 | 104 | 108 | 120 | 100 | 108 | 104 | 108 | 108 | 116 | 116 | 100 | 100 | 106 | ---- | ---- | ---- | ---- | ---- | ---- | ---- | ---- | ---- | ---- | ---- | ---- | ---- | ---- | ---- | ---- | Uncomplicated. Recovered. |
| | | T. | ---- | 106.3 | 104.5 | 105.5 | 104 | 105 | 104.5 | 103.5 | 102 | 105 | 102.5 | 106 | 104 | ---- | 103 | 104 | 100 | 104 | 101.5 | 105 | ---- | ---- | 102 | 104 | 102.5 | ---- | 103.5 | 105.5 | 102.5 | 105.3 | 101.5 | 105 | 101 | 104.5 | 103 | 105.5 | 102 | 105 | 101 | 104 | ---- | ---- | ---- | ---- | ---- | ---- | ---- | ---- | ---- | ---- | ---- | ---- | ---- | ---- | ---- | ---- | |
| | | R. | ---- | 38 | 32 | 38 | 25 | 40 | 28 | 27 | 32 | 28 | 25 | 32 | 28 | ---- | 27 | 28 | 24 | 28 | 24 | 28 | ---- | ---- | 32 | 36 | 25 | ---- | 20 | 28 | 28 | 32 | 24 | 30 | 28 | 30 | 32 | 32 | 36 | 24 | 20 | 22 | ---- | ---- | ---- | ---- | ---- | ---- | ---- | ---- | ---- | ---- | ---- | ---- | ---- | ---- | ---- | ---- | |
| 24 | 8th | P. | 108 | 108 | 112 | 112 | 108 | 128 | 96 | 104 | 104 | 120 | 104 | 120 | 100 | 104 | 100 | 116 | 110 | 112 | 108 | 100 | 100 | 112 | 100 | 116 | 100 | ---- | 96 | 92 | 96 | 118 | 84 | 100 | 96 | 100 | 100 | 112 | 82 | 100 | 88 | 100 | 96 | 100 | 100 | 90 | 90 | 100 | 100 | 98 | 96 | 90 | 92 | ---- | ---- | ---- | ---- | ---- | Pneumonia. Recovered. |
| | | T. | 105 | 105 | 105 | 105 | 105.5 | 106 | 104 | 104.5 | 102.7 | 104.5 | 102.8 | 104 | 102 | 104 | 102.3 | 103.3 | 100.5 | 104 | 100 | 102 | 102.6 | 103.5 | 99.3 | 104.7 | 100.7 | ---- | 101 | 102.7 | 101.6 | 104 | 98 | 103 | 100.3 | 102.7 | 101.7 | 103.3 | 97.7 | 103.7 | 99.3 | 102.3 | 98.5 | 100.3 | 103.5 | 100.3 | 101.7 | 101 | 102.5 | 100.3 | 100.2 | 100.5 | 100 | ---- | ---- | ---- | ---- | ---- | |
| | | R. | 24 | 28 | 32 | 36 | 28 | 32 | 28 | 30 | 48 | 44 | 56 | 50 | 44 | 55 | 60 | 50 | 40 | 42 | 50 | 40 | 44 | 60 | 36 | 36 | 36 | ---- | 40 | 36 | 36 | 50 | 32 | 32 | 36 | 30 | 36 | 32 | 28 | 36 | 34 | 34 | 30 | 36 | 36 | 28 | 26 | 28 | 24 | 23 | 22 | 24 | 20 | ---- | ---- | ---- | ---- | ---- | |

Tabulated Statement of Eight Cases of Remittent Fever.

| No. of Patient. | Day of disease on which observation began. | Pulse, Temp'ture, Respirati'n | 1st Day. | | 2d Day. | | 3d Day. | | 4th Day. | | 5th Day. | | 6th Day. | | 7th Day. | | 8th Day. | | 9th Day. | | 10th Day. | | 11th Day. | | 12th Day. | | 13th Day. | |
|---|---|---|---|---|---|---|---|---|---|---|---|---|---|---|---|---|---|---|---|---|---|---|---|---|---|---|---|---|
| | | | M. | E. | M. | E. | M. | E. | M. | E. | M. | E. | M. | E. | M. | E. | M. | E. | M. | E. | M. | E. | M. | E. | M. | E. | M. | E. |
| 1 | 1st. | P. | ----- | 90 | 84 | 96 | 90 | 120 | 98 | 112 | 80 | 86 | 76 | 74 | 100 | 116 | 104 | 116 | 96 | 86 | 84 | 80 | 72 | 76 | 72 | 74 | 72 | ----- |
| | | T. | ----- | 103.5 | 102.8 | 103.5 | 103 | 105 | 102.5 | 103.7 | 99.5 | 100.5 | 99.5 | 99.3 | 103 | 103.5 | 102 | 104 | 101.7 | 100.3 | 100.5 | 98.7 | 98.5 | 99.5 | 98.5 | 99.3 | 98.5 | ----- |
| | | R. | ----- | 23 | 20 | 24 | 22 | 30 | 24 | 28 | 18 | 20 | 18 | 18 | 28 | 30 | 25 | 28 | 24 | 20 | 18 | 18 | 18 | 20 | 18 | 18 | 18 | ----- |
| 2 | 3rd. | P. | ----- | 84 | 80 | 88 | 80 | 88 | 76 | 88 | 80 | 82 | 74 | 76 | ----- | ----- | ----- | ----- | ----- | ----- | ----- | ----- | ----- | ----- | ----- | ----- | ----- | ----- |
| | | T. | ----- | 103.3 | 100.7 | 103.3 | 100.5 | 101.7 | 98.8 | 102 | 99.5 | 101 | 99 | 100 | ----- | ----- | ----- | ----- | ----- | ----- | ----- | ----- | ----- | ----- | ----- | ----- | ----- | ----- |
| | | R. | ----- | 24 | 20 | 24 | 24 | 28 | 22 | 28 | 22 | 24 | 20 | 20 | ----- | ----- | ----- | ----- | ----- | ----- | ----- | ----- | ----- | ----- | ----- | ----- | ----- | ----- |
| 3 | 5th. | P. | ----- | 96 | 96 | 104 | 90 | 86 | 90 | 100 | 80 | 96 | 72 | 74 | 72 | 72 | ----- | ----- | ----- | ----- | ----- | ----- | ----- | ----- | ----- | ----- | ----- | ----- |
| | | T. | ----- | 105 | 104 | 103 | 103 | 100 | 102 | 103 | 101.5 | 102.5 | 100 | 100 | 98.5 | 99 | ----- | ----- | ----- | ----- | ----- | ----- | ----- | ----- | ----- | ----- | ----- | ----- |
| | | R. | ----- | 30 | 28 | 28 | 30 | 28 | 28 | 24 | 24 | 24 | 18 | 18 | 18 | 18 | ----- | ----- | ----- | ----- | ----- | ----- | ----- | ----- | ----- | ----- | ----- | ----- |
| 4 | 5th. | P. | ----- | 96 | 100 | ----- | 90 | 80 | 92 | 96 | 80 | 96 | 92 | 90 | 84 | 76 | ----- | ----- | ----- | ----- | ----- | ----- | ----- | ----- | ----- | ----- | ----- | ----- |
| | | T. | ----- | 103 | 104.5 | ----- | 103 | 100 | 102.7 | 103.5 | 102.5 | 105 | 101 | 100 | 99 | 98.5 | ----- | ----- | ----- | ----- | ----- | ----- | ----- | ----- | ----- | ----- | ----- | ----- |
| | | R. | ----- | 24 | 28 | ----- | 28 | 20 | 20 | 20 | 20 | 24 | 18 | 20 | 18 | 18 | ----- | ----- | ----- | ----- | ----- | ----- | ----- | ----- | ----- | ----- | ----- | ----- |
| 5 | 8th. | P. | 96 | 100 | 88 | 90 | 90 | 80 | ----- | ----- | ----- | ----- | ----- | ----- | ----- | ----- | ----- | ----- | ----- | ----- | ----- | ----- | ----- | ----- | ----- | ----- | ----- | ----- |
| | | T. | 106 | 104 | 103.5 | 101 | 100 | 98.5 | ----- | ----- | ----- | ----- | ----- | ----- | ----- | ----- | ----- | ----- | ----- | ----- | ----- | ----- | ----- | ----- | ----- | ----- | ----- | ----- |
| | | R. | 56 | 30 | 16 | 16 | 18 | 16 | ----- | ----- | ----- | ----- | ----- | ----- | ----- | ----- | ----- | ----- | ----- | ----- | ----- | ----- | ----- | ----- | ----- | ----- | ----- | ----- |
| 6 | 5th. | P. | ----- | 76 | 50 | 76 | 66 | 72 | 72 | ----- | ----- | ----- | ----- | ----- | ----- | ----- | ----- | ----- | ----- | ----- | ----- | ----- | ----- | ----- | ----- | ----- | ----- | ----- |
| | | T. | ----- | 104 | 101 | 103 | 100 | 102.5 | 98.5 | ----- | ----- | ----- | ----- | ----- | ----- | ----- | ----- | ----- | ----- | ----- | ----- | ----- | ----- | ----- | ----- | ----- | ----- | ----- |
| | | R. | ----- | 24 | 18 | 20 | 16 | 16 | 18 | ----- | ----- | ----- | ----- | ----- | ----- | ----- | ----- | ----- | ----- | ----- | ----- | ----- | ----- | ----- | ----- | ----- | ----- | ----- |
| 7 | 9th. | P. | 96 | 104 | 90 | 124 | 104 | 92 | 90 | ----- | ----- | ----- | ----- | ----- | ----- | ----- | ----- | ----- | ----- | ----- | ----- | ----- | ----- | ----- | ----- | ----- | ----- | ----- |
| | | T. | 99.3 | 105 | 99.3 | 105 | 97 | 98.5 | 99 | ----- | ----- | ----- | ----- | ----- | ----- | ----- | ----- | ----- | ----- | ----- | ----- | ----- | ----- | ----- | ----- | ----- | ----- | ----- |
| | | R. | 17 | 24 | 16 | 24 | 16 | 16 | 16 | ----- | ----- | ----- | ----- | ----- | ----- | ----- | ----- | ----- | ----- | ----- | ----- | ----- | ----- | ----- | ----- | ----- | ----- | ----- |
| 8 | 7th. | P. | ----- | 72 | 70 | 70 | 68 | 70 | 64 | 68 | ----- | ----- | ----- | ----- | ----- | ----- | ----- | ----- | ----- | ----- | ----- | ----- | ----- | ----- | ----- | ----- | ----- | ----- |
| | | T. | ----- | 104 | 102.3 | 103 | 98.5 | 103 | 99 | 101.5 | ----- | ----- | ----- | ----- | ----- | ----- | ----- | ----- | ----- | ----- | ----- | ----- | ----- | ----- | ----- | ----- | ----- | ----- |
| | | R. | ----- | 24 | 20 | 20 | 24 | 20 | 24 | 20 | ----- | ----- | ----- | ----- | ----- | ----- | ----- | ----- | ----- | ----- | ----- | ----- | ----- | ----- | ----- | ----- | ----- | ----- |

or pain in the head; and those who had nose-bleed almost invariably expressed relief from the headache after the bleeding. From the almost uniform presence of headache and flushed face in the early stage of typhoid fever, there seems to be a passive congestion of the brain and whole head, and the hemorrhage occurs from a rupture of the vessels of the schniderian membrane from over-distension. Though epistaxis often recurs repeatedly in the first week of the fever (in some of the cases we have recorded, as often as five or six times daily), it is not apt to be abundant, and seldom if ever calls for treatment. It is different when it occurs in the second or third week. It is then due to an acute hemorrhagic diathesis, and may require special treatment. Though the hemorrhage was not excessive in either of the three cases we observed, the fever was very severe, and two of the cases were fatal.

*Diarrhœa.* This is the most constant symptom in typhoid fever except the increase of temperature, but it is not usually one of the earliest symptoms, and taken alone is of little importance in point of diagnosis. The character and odor of the discharges are of importance, and when they present that badly cooked pea-soup appearance and that peculiar odor, more easily recognized than described, are pathognomonic. The diarrhœa is due to the catarrhal inflammation, said to be specific, which so universally affects the mucous surfaces in typhoid fever. We may moderate it by treatment but we cannot check it entirely, and it is not desirable if we could, for it is the experience of all, I believe, that a lax condition of the bowels is most favorable in this disease.

We have stated that diarrhœa was present in 77 of the 112 cases. It was present in this number in a degree to call for treatment, and in 25 more the bowels were free. As a rule, treatment was not resorted to where there were not more than four or five stools daily. The remedies first given were, olei terebinth and tincture opii in emulsion, the formula generally used being: Olei terebinth, dr. j; Tr. opii, dr. j; emuls. ad oz. j and dr. j doses three or four times daily. This emulsion controlled the diarrhœa in nearly all the cases. Argenti nitratis, cupri sulph., or some other astringent, was resorted to in a few cases.

*Constipation.* We have placed this along with symptoms, from lack of a better place for it. In exceptional cases, constipation is present in typhoid fever. It was present in 10 of the 112, but in no case was it obstinate. It readily yielded to mild treatment, but it was necessary to repeat the laxative a number of times in each case. The cases in which it occurred were mostly mild, but it was observed that when the bowels were allowed to go unmoved over three days, there was a slight increase of the temperature and an aggravation of other symptoms. If the constipation continued longer than this, a small dose of castor oil was given, and if this did not act, an injection was ordered; no case required more than this, and usually the oil was sufficient.

*Rose Spots.* These were observed in 50 of the cases under consideration, and possibly occurred in a larger proportion, as there was a goodly number of the 112 that did not come under observation until the third or fourth week of the disease, late enough for the rash to have been present and disappeared. This eruption has been given a wide range of importance by different observers, some making it *the* characteristic of typhoid fever, others barely mentioning it as a symptom. Perhaps this diversity of opinion has mainly arisen from the varying proportion of cases in which it is present in different epidemics and in different places. Trousseau says: "In some localities, as at Paris, the spots are found with sufficient constancy to justify our looking out for them as the most obvious pathognomonic sign, but there are other places in which attentive observers have never been able to see them." Flint observed the rash in 49 of 73 cases, but says that he has observed the spots in other diseases. If all do not admit that this eruption is peculiar to typhoid, I believe none deny that it is a sign of much diagnostic importance.

In the 50 of the 112 cases in which the spots were present, in four the rash began to appear the first week, 38 in the second week, and eight in the third week of the disease. The earliest it was observed to appear was on the fifth day, the latest on the eighteenth; in the greatest number of cases on the twelfth day. The duration of the rash was not noted, and it was not observed that it bore any relation to the severity of the disease. It was present in a number of the mildest cases and absent in many of the severest. Of the 20 fatal cases, it was only observed in 7, but as the majority of these were admitted late in the disease, it might have been present in a greater number of them. Successive crops of these spots have been observed to appear in the same patient Trousseau relates a case in which there were three separate eruptions. This reappearance of the rash was not observed in any of the 50.

*Blue Spots.* These were only found in two cases, and I am inclined to think are, in epidemics in this country, of more interest as a rarity than importance as a sign. Trousseau speaks of the blue spots as not uncommon, but he had only observed them in very mild cases. In both cases we observed the spots appeared about the tenth day and ran a course in every way similar to the rose rash. One of the cases was mild, but the other was quite severe. I am not aware that these spots are ever found in any but typhoid cases. In the eruption we observed, there was no elevation of the surface, but simply spots of a pale blue color appearing beneath the cuticle, irregular in outline and varying in size from a three to a ten cent piece.

*Temperature.* Increase of temperature is the only symptom present in every case of typhoid fever, and we believe its rise and course are pathognomonic of the disease. When we are unable, in the first two weeks of the fever, to make a diagnosis from other symptoms, a few observations with the thermometer will enable us to say positively whether we have a case of typhoid or not.

Thierfelder first called attention to the use of the thermometer in this disease, but Wunderlich, by his many observations and valuable deductions, has done more than any other man to bring it prominently before the profession. We have tabulated twenty-four cases, which will show at a glance the record of the pulse, temperature and respirations in each. The record in each case was not run entirely through to convalescence, but in a number only continued until the acme was passed. We will let the table speak for itself. Perhaps we cannot more forcibly present the importance of the thermometer, as a means of diagnosis in this disease, than by relating the following history: C. P., aged 17 years; servant; Dane: admitted into hospital November 16, 1872. The patient was unable to speak English, but through an interpreter we learned that she arrived from her native country six weeks previous, and that she had had good health up to that time. About the time she landed in this country she began to cough, and this had continued more or less ever since, but she was able to be up until two days before. This was all we were able to learn of her previous history. On examination we found the patient in good flesh—intellect clear; face flushed; skin hot and dry; tongue slightly coated, white, and a little dry; appetite poor, but not much thirst; bowels regular; abdomen a little full, and tender on pressure; gurgling in right iliac fossa. Cough is not severe, but the patient expectorates considerable purulent matter; marked dullness over upper half of right lung; cavernous murmur in right infraclavicular region. Of course, it was decided that the patient had phthisis, and was not this sufficient to account for all her symptoms so far? Surely there was nothing thus far to warrant us in saying she had typhoid fever also. True, she had a little fullness and tenderness of the abdomen, and gurgling in the right iliac fossa, but these we may get in diarrhœa of phthisis, and, besides this, typhoid fever rarely occurs in consumptives. A record of the temperature was kept, which may be seen by reference to the table, case 15, and from this alone a positive diagnosis of typhoid fever was made, and Dr. Ross presented the patient at his clinic for the purpose of speaking of the complication.

The case ran along through a severe course of the fever without much change in the pulmonary trouble, but as soon as she began to convalesce from the fever, the tubercle rapidly increased, and January 21, 1873, the patient died. A post-mortem examination showed the right lung almost gone, and the left stuffed with tubercle. There was no tubercular disease of Peyer's patches, but there were cicatrices, which showed unmistakable evidence of recent extensive typhoid lesions. We can scarcely conceive of a case presenting stronger evidence in favor of the thermometer in diagnosis than does this case. But where we found the thermometer most practically useful was in distinguishing between remittent and typhoid fevers. I believe it is often utterly impossible, in the early stages of these fevers, to distinguish between them without the use of the thermometer, and I believe, too, that many cases of remittent are called typhoid, and that the wondrous success of some physicians in the treatment of typhoid is thus explained. For the purpose of showing the contrast in the course of the temperature of these two fevers, we have tabulated a few cases of remittent also. It will be seen from this, that while the morning temperature is usually the highest, there is no uniformity in its course. It seldom continues above 103° over forty-eight hours, and when the remission occurs the fall is sudden, as also the rise if the exacerbation returns. To further illustrate the course of the temperature in the two diseases, we have drawn the following diagram (diagram omitted for want of space) from case 1 of the remittent and case 13 of the typhoid cases.

The thermometer is not alone useful as an aid in diagnosis in typhoid fever; it is of equally as much importance in prognosis. It will be observed, by reference to the table of typhoid cases, that the deductions to be drawn agree in the main with those of Wunderlich. The temperature rises gradually during the first week, and continues much the same into the third week, when, in favorable cases, morning remissions begin to occur; and from the middle of this week the evening temperature begins to descend also. From a lack of early observation, we were unable to fully verify what Wunderlich states to be a rule, that the temperature rises two degrees toward evening and falls one degree before next morning; but of this we have no doubt, and have drawn our diagram to accord with it. With reference to the diagnosis, Wunderlich says, when the temperature rises to 104° by the first or second day of the attack, the disease is not typhoid fever; and when, by the evening of the fourth day, the temperature has not reached 103°, the disease is not typhoid. As regards prognosis, the more important deductions are:

*First.* If the temperature does not rise above 105° during the second week, and begins to decline by the beginning of the third week, the prognosis is favorable.

*Second.* When the temperature rises above 106°, and is maintained as high as 105° into the third week, the prognosis is very unfavorable.

*Third.* If the temperature rises as high as 107°, the case is almost surely fatal. In six of the cases we observed, the temperature rose as high as 107°, and not one of the six recovered.

*Fourth.* Marked irregularities occurring during the second week demand attention.

*Fifth.* Convalescence is not completely established until the evening temperature has reached the normal standard.

As in this disease the greatest danger is from the severity of the fever, the reduction of the temperature becomes an important consideration. According to Niemeyer, the excessive bodily heat produces death by causing paralysis of the heart, and a high temperature, long continued, produces death by consuming the body of the patient. Numerous means have been resorted to for the purpose of reducing the temperature in this disease, but we will only mention those used in the cases under consideration.

In all of these cases, iced and acidulated drinks were allowed freely, and the sponge-bath was ordered twice daily when the fever was high. These were not only grateful to the patients, but had a decided influence in reducing the temperature.

Quinine was administered in a number of cases, with the effect apparently of lowering the temperature. This effect was most marked in cases which, in the second week of the disease, had a difference of as much as 3° in the morning and evening temperature—say 103° in the morning and 106° in the evening. We did not observe so great a difference in but few cases, and are inclined to think it was due to a malarial element. Any way, quinine in gr. v doses three times daily seemed to lessen the evening rise.

The cold bath was only resorted to in two cases, and in neither of these until the temperature had reached 107°. The patients with this temperature were put in the bath at 90°, and kept in fifteen minutes, during which time the bath was lowered to 80°. The temperature of the patient in each case was reduced 6° by the first bath, and the delirium was quieted for a time. The bath was repeated a number of times with similar results, but both cases terminated fatally about the end of the second week. While the bath did not save these desperate cases, it is certainly a very efficient means of reducing the temperature, and, it seems to us, is preferable to packing in wet sheets.

The pulse and respirations, though not of so much importance as the temperature in this disease, are of sufficient importance to demand recording. It will be seen by referring to the table that both bear some relation to the temperature, but do not run so uniform a course. The pulse is the best guide for the use of stimulants, and the rule was, in the treatment of these cases, to begin to stimulate as soon as the pulse became soft and compressible. A pulse of 120 is about as unfavorable as a temperature of 106°, and of 130 as a temperature of 107°.

The respirations are of most value in indicating the severity of the bronchial catarrh so often present in this disease.

*Complications—Delirium.* This we have said was present in 49 of the 112 cases. It is usually most marked in severe cases, and may arise at any time in the course of the fever, and it may take on any form, from the low muttering to the very violent.

As delirium rapidly exhausts the patient, it is of the utmost importance to control it if possible. Sir Wm. Gull says that it is due to *irritation* of the brain, and not, as some have imagined, to inflammation, and that the one great remedy for this irritation is alcohol. Irritation is doubtless the cause of the delirium, but we did not observe any benefit from the use of alcohol in this complication, though it was administered in the majority of the cases. The great remedy in the treatment of delirium and sleeplessness in these cases was the hydrate of chloral.

Not all of the cases in which delirium occurred needed special treatment, but a number who had no delirium were unable to sleep. Chloral was administered in forty-eight cases, in which one or the other of these were present, and in only five cases did it fail to quiet and produce sleep. It was ordered in gr. xv doses every fifteen minutes, beginning at bed-time, and continuing until sleep was produced or three doses were taken. If the first batch failed, as it sometimes did, after a few hours it was repeated again. No bad effect was observed from its use in this way, and, as we have said, there were only five complete failures. The sleep usually continued from three to six hours. In the worst cases the delirium returned with waking, but was less violent each succeeding time the treatment was given. Before the three-dose rule was adopted, one patient was given the fourth dose, when he fell into a heavy sleep, which lasted nearly twenty-four hours, but he finally roused up and went on to a rapid recovery. From the experience we have had with this remedy, we have no hesitancy in saying that there is *no medicine* used in the treatment of typhoid fever that we would not sooner give up than hydrate of chloral.

*Deafness.* This, as we have said, was present in eleven cases. It usually appears in the second week of the fever, and when it occurs on both sides, is probably produced by a propagation of the typhous catarrh to the eustachian tube and cavity of the tympanum. This complication does not usually require any special treatment, and has been mostly spoken of with reference to its prognostic import, though its significance has been very differently regarded. Trousseau regards deafness on both sides as favorable, but when on one side, as unfavorable. In all of the cases in which we observed deafness, the fever was very severe, and three of the cases were fatal. In one case which recovered, there was suppuration of the middle ear and perforation of the membrane of the tympanum; but this and similar cases I believe to be the result of a genuine inflammation, and, when detected, should have the same treatment as an ordinary acute inflammation under other circumstances. When it occurs in severe cases of fever, as was this case, the inflammation may not be suspected until its results are seen; but if the patient is sensible of pain, we are pretty sure to learn of it as soon as it presents. If it is discovered early, local blood-letting, or a continuous stream of warm water directed into the external meatus, will often arrest it. This trouble should certainly never be neglected when it is discovered. In all of the other cases which recovered, the hearing was perfectly restored when convalescence was complete.

*Softening of the Cornea.* This is not peculiar to typhoid fever, and only occurred in one of these cases. It results principally, if not entirely, from the constant exposure of the eye to the air through want of power to wink or close the eye while sleeping. Trousseau, acting upon this supposition, introduced the very simple but efficient treatment of mechanically closing the eyes. In this case the lids were closed by means of adhesive strips, and the cornea very soon cleared up. If this complication is neglected, rapid destruction of the eye may result, but certainly so common-sense an item of treatment will not be forgotten.

*Parotitis* occurred in three cases. This complication has generally been regarded as a very grave one. In the three cases it came on in the fourth week of the fever, and, in two of them, both glands were involved, and suppurated. Both of these recovered, but the other, in which one gland was involved, was fatal. The treatment was, early poulticing, and anodynes sufficient to control the pain. As soon as fluctuation was detected, a free opening was made, and though the discharge was free, it did not continue longer than from an ordinary abscess. In the two cases which recovered, convalescence was not delayed beyond the usual time of severe cases of typhoid fever.

*Intestinal hemorrhage* occurred in three cases. Though this has generally been regarded as a grave complication, the two greatest clinical teachers, Graves and Trousseau, have regarded it otherwise. They have even expressed the opinion that it is usually of favorable augury. All three of the cases we observed were fatal. In two, the hemorrhage occurred in the third week of the fever, and was the direct cause of death. One of these did not have any escape of blood, externally, but suddenly went into collapse and died. A *post-mortem* examination showed a large quantity of blood in the colon and lower portion of the ileum. It was found that the hemorrhage had resulted from an erosion of vessels, caused by the separation of a slough from a small but deep ulcer about two feet above the ileo-cæcal valve. The other of these two cases lost a large-sized chamberful of blood during the twenty-four hours preceding death. A number of styptics were administered, but did not arrest the hemorrhage until, finally, the fluid extract of ergot in dr. ss. doses every half hour was ordered, and the bleeding soon ceased. In the third case, the hemorrhage first occurred in the second week, and kept recurring at intervals until the death of the patient on the thirty-fifth day. The obstinacy of friends prevented any autopsy of either this or the preceding case.

*Peritonitis* occurred in two cases, though perforation did not occur in either. One of these cases presented peritonitis for a week or more before death, which occurred in the sixth week of the fever. On opening the abdomen, in the autopsy of this case, the intestines were found to be quite firmly attached to each other at a number of points, and, in one point, to the peritoneum lining the right iliac fossa. On making slight traction, a number of these patches of attachment separated, leaving openings in the intestines from one-half to three-quarters of an inch in diameter. There was no obstruction of the bowels, and the patient died of exhaustion after the inflammation had subsided. The other patient died in the fifth week of the fever, but did not present any symptoms of peritonitis until a few hours before death. On opening the abdomen of this case, the whole peritoneal surface presented a congested appearance, which was very marked in the region of the spleen. This organ was very large, and presented an almost gangrenous appearance. The peritoneal cavity contained about half a pint of bloody serum. Extensive ulcerations of Peyer's patches and the solitary glands were found, also considerable enlargement of the mesenteric glands. The treatment was, opium sufficient to control pain, and the turpentine emulsions three or four times daily, with turpentine stupes to the abdomen.

*Sequelæ.* Of these we have only noticed two, though others of less interest occurred. Right hemiplegia, with aphasia, was met with in one case, and we mention it because it was of considerable interest to us. It occurred in an intelligent patient on the twenty-first day of a severe attack of fever. The patient had begun to improve, but on this day had a severe chill, which lasted three or four hours. By means of stimulants and artificial heat a reaction was finally brought about, but when the chill had passed off the patient's right side was paralyzed, and he was unable to speak, though he evidently knew what was said to him, and tried to speak. In a few days the patient began to move the extremities a little, and answer *yes* to everything said to him. He remained in the hospital until the fifty-fourth day from the attack of fever, and had gradually added to his vocabulary until he could speak short sentences intelligently. The paralysis had improved very much, but the patient was yet unable to walk. Of course, the paralysis and aphasia were most probably caused by an embolus plugging an artery in the left hemisphere of the cerebrum. In addition to tonics and nutritious diet, the patient was ordered a solution of the iodide of potassium and bichloride of mercury, on the theory that these remedies favor absorption.

*Œdema of Left Lower Extremity.* Dropsy or œdema of some part of the body is not an uncommon sequel of typhoid fever, but was only met with in three of these cases, and in all three occurred in the left lower extremity. In each case the œdema came on in a similar manner after convalescence appeared to be established. The patients first complained of severe pain in the left groin, but after a day or two the whole extremity became more or less painful, and began to swell. When the œdema became excessive, as it did in each case, the limb presented an appearance very similar to that of phlegmasia dolens. The cause of the œdema was doubtless plugging of the femoral veins by thromboses. Its occurrence in the left extremity in each case was perhaps a coincidence. The only special treatment consisted in elevating the limb and enveloping it in cotton and oil-silk. Two of the cases were under observation for a number of weeks, and though the œdema had subsided very much, there was still considerable swelling and stiffness when the patients were lost sight of.

*Mortality.* This, according to different authors, varies from one death in twenty to one in four. Of the 112 cases twenty were fatal, or, one death in five and three-fifth cases. Of the twenty fatal cases, six were moribund on admission, and died a few hours afterward. All of these were severe cases, and had reached the third week of the disease. I do not know that I will have a better opportunity than at this point to enter a protest against the *inhumanity of removing typhoid fever patients after they have reached this stage of this disease.* I do not remember an instance in which a severe case of typhoid was brought into the hospital in the third or fourth week, and recovered. Patients that have advanced so far in the disease, even though it be severe, and the scale seems to waver between life and death, usually recover with proper care. But let them be bundled into an express wagon, or an ambulance even, and driven from one to four miles, and it is very evident which way the scale will turn. They might just as well be knocked on the head and taken to the dead-house direct, so far as results are concerned. Common humanity certainly demands that these patients be provided for where they are found, that they may have, at least, a chance for their lives.

Of the fourteen remaining fatal cases, only six were uncomplicated. All of these died between the twelfth and seventeenth days.

The average duration of the disease, in all of the fatal cases, was about twenty-three days. In the cases which recovered, the average duration was thirty-two days.

Of the whole number of cases, ninety-four were males and eighteen females, and of the fatal cases, sixteen were males and four females.

A word in conclusion with regard to general treatment and diet. As soon as the diagnosis of typhoid fever was made, the patient was put upon the following solution :

R. Potassæ Chloratis, - - - - dr. ij.<br>

Acidi Muriatici, dil., - - - oz. ss.<br>

Syrupi Simplex, - - - - - - oz. j. ss.<br>

Aquæ, - - - - - - - oz. iv.<br>

M. S. Tablespoonful every three hours in water.

This was continued until the severity of the fever was past, and in mild cases no other medicine was given. When patients began to need stimulants they were first ordered milk punch or wine whey, and if this was not sufficient, the following solution was ordered :

R. Strychniæ Sulphatis, - - - - - gr. j.<br>

Acidi Nitrici, - - - - - - dr. j.<br>

Syrupi Simplex,<br>

Aquæ, - - - - - - *aa* oz. ij.<br>

M. S. Teaspoonful once in six hours.

When active stimulation was demanded, a solution of quinine and whisky, gr. ij to the oz. j was ordered in oz. ss. doses as often as required. The patients were kept as quiet as possible, and strict attention was paid to cleanliness. It was made the imperative duty of the nurses to empty all discharges as soon as passed, and disinfectants—carbolic acid, chloride of lime and bromine—were used freely. There was not a single case of typhoid fever developed in the hospital during the treatment of these cases, though they were mixed in promiscuously with other patients. I believe that the thorough cleanliness and disinfection account for it.

Owing to the crowded condition of the hospital, one of the most important agents in the treatment of the disease—*pure air*—was deficient. Each patient only had between 700 and 900 cubic feet of space, not more than half as much as they ought to have had. The diet, though nourishing, was strictly fluid. Beef-tea, milk, broth and soups were given in abundance and as regularly as medicines. This diet was continued until the evening temperature had returned to the normal standard.

